# CHIP ameliorates neuronal damage in H_2_O_2_-induced oxidative stress in HT22 cells and gerbil ischemia

**DOI:** 10.1038/s41598-022-22766-0

**Published:** 2022-11-30

**Authors:** Kyu Ri Hahn, Hyun Jung Kwon, Yeo Sung Yoon, Dae Won Kim, In Koo Hwang

**Affiliations:** 1 Department of Anatomy and Cell Biology, College of Veterinary Medicine, and Research Institute for Veterinary Science, Seoul National University, Seoul, 08826 South Korea; 2 Department of Biochemistry and Molecular Biology, Research Institute of Oral Sciences, College of Dentistry, Gangneung-Wonju National University, Gangneung, 25457 South Korea; 3 Department of Biomedical Sciences, and Research Institute for Bioscience and Biotechnology, Hallym University, Chuncheon, 24252 South Korea

**Keywords:** Neuroscience, Anatomy

## Abstract

Carboxyl terminus of Hsc70-interacting protein (CHIP) is highly conserved and is linked to the connection between molecular chaperones and proteasomes to degrade chaperone-bound proteins. In this study, we synthesized the transactivator of transcription (Tat)-CHIP fusion protein for effective delivery into the brain and examined the effects of CHIP against oxidative stress in HT22 cells induced by hydrogen peroxide (H_2_O_2_) treatment and ischemic damage in gerbils by 5 min of occlusion of both common carotid arteries, to elucidate the possibility of using Tat-CHIP as a therapeutic agent against ischemic damage. Tat-CHIP was effectively delivered to HT22 hippocampal cells in a concentration- and time-dependent manner, and protein degradation was confirmed in HT22 cells. In addition, Tat-CHIP significantly ameliorated the oxidative damage induced by 200 μM H_2_O_2_ and decreased DNA fragmentation and reactive oxygen species formation. In addition, Tat-CHIP showed neuroprotective effects against ischemic damage in a dose-dependent manner and significant ameliorative effects against ischemia-induced glial activation, oxidative stress (hydroperoxide and malondialdehyde), pro-inflammatory cytokines (interleukin-1β, interleukin-6, and tumor necrosis factor-α) release, and glutathione and its redox enzymes (glutathione peroxidase and glutathione reductase) in the hippocampus. These results suggest that Tat-CHIP could be a therapeutic agent that can protect neurons from ischemic damage.

## Introduction

Brain ischemia is one of the most prevalent neurological disorders associated with aging, and approximately 80% of stroke events are known as ischemic strokes^1^. Interruption of blood supply to the brain in ischemic strokes causes massive neuronal loss in the brain, including the hippocampus, resulting in severe complications and reduced quality of life^2^. Various cell death mechanisms have been proposed, including oxidative stress induced by reactive oxygen species (ROS) and neuroinflammation induced by pro-inflammatory cytokines, such as interleukin (IL)-1β, IL-6, and tumor necrosis factor-α (TNF-α)^3–5^. However, few therapeutics are available, except for tissue plasminogen activator, because of their low bioavailability and permeability to the blood–brain barrier.

The blood–brain barrier is a highly selective barrier formed by endothelial cells, astrocyte end-feet, and pericytes^6^. Only small and highly lipophilic peptides can cross this barrier^7^. Many attempts have been made to facilitate the delivery of proteins into brain tissues^8,9^. Human immunodeficiency virus-1 (HIV-1) transactivator of transcription (Tat), one of the most well-known protein transduction domains, originates from the HIV-1 virus and crosses the plasma membrane in a receptor- and transporter-independent manner^10,11^. In previous studies, we observed that Tat-fused proteins could deliver target proteins into intracellular spaces in HT22 cells and into the hippocampus^12,13^.

Carboxyl terminus of Hsc70-interacting protein (CHIP), encoded by the *STUB1* gene, exhibits high similarity of amino acid sequences between rodents and humans and is ubiquitously expressed in muscle and nervous tissues^14^. It consists of triple tandem tetratricopeptide repeat domains in the N-terminus, which interact with chaperones such as heat shock protein 70/90, and a U-box domain at the C-terminus which has E3 ubiquitin ligase activity^14–16^. Based on its structure, CHIP plays an important role in the connection between molecular chaperones and proteasomes to degrade chaperone-bound proteins^17–19^. Evidence has shown that CHIP is closely related to the pathophysiology of ischemia^20–24^, although its underlying mechanisms are not fully understood. In addition, there has been conflicting evidence stating that CHIP has neuroprotective and detrimental effects against ischemic damage^21,22,24,25^. For example, CHIP-overexpressing cells worsen oxidative stress and ubiquitination of proteins, and downregulation of CHIP by siRNA in primary neuronal cultures ameliorates the neuronal damage induced by oxidative stress^21^. However, CHIP protects neurons from oxygen-glucose deprivation^24^, whereas CHIP-deficient neurons are more susceptible to oxygen-glucose deprivation^22,25^.

Mongolian gerbils were used in animal models for transient forebrain ischemia because they have incomplete posterior communicating arteries^26^. Ligation of the common carotid artery easily induces transient forebrain ischemia, and the procedures of ischemic surgery are more convenient and quicker to induce ischemia compared to models for middle cerebral artery occlusion in mice and rats^27^. In addition, ischemic gerbils survive for longer than 1 year, and it is easy to conduct follow-up study for neuronal and glial regeneration in the hippocampus^28^. A recent study demonstrated the successful generation of knock-out gerbils based on CRISPR/Cas9 technology, which facilitated the study using gerbils^29^.

In the present study, we synthesized a Tat-CHIP fusion protein to facilitate the delivery of proteins in HT22 cells and the gerbil hippocampus. We examined the effects of Tat-CHIP on cell survival in two different paradigms, H_2_O_2_-induced oxidative stress in HT22 cells and transient forebrain ischemia in gerbils, to elucidate the roles of Tat-CHIP in oxidative and ischemic damage.

## Materials and methods

### Synthesis of Tat-CHIP and its control protein

Polymerase chain reaction (PCR) was performed to amplify 10 ng human CHIP cDNA (accession number: NM_005861) (Takara Bio Inc., Shiga, Japan) as a template using the sense (5′-CTCGAGATGAAGGGCAAG-3′) and antisense (5′-GGATCCTCAGTAGTCCTC-3′) primers. Sequential reactions were performed at 94 °C for 5 min and 30 s to denature DNA, 51 °C for 30 s to anneal primers, and 72 °C for 40 s to extend DNA. The cycles were repeated 30 times and final extension was performed at 72 °C for 5 min. The final CHIP PCR product was obtained at 912 bp in size. Thereafter, the PCR product was cloned into the Tat expression vector containing a polyhistidine-tag, as described previously^30^. Tat-CHIP and its control CHIP plasmids were cloned into the *Escherichia coli* strain BL21 (DE3) cells, and overexpression of proteins was induced by incubating with 0.5 mM isopropyl-β-D-thiogalactoside (IPTG, Duchefa, Haarlem, the Netherlands) for 5 h. Induction efficiency of Tat-CHIP by IPTG was assessed with various concentrations of IPTG (0.25, 0.5, 0.75, and 1.0 mM), and the optimal concentration was 0.5 mM for Tat-CHIP. Subsequently, the purification and desalting of Tat-CHIP and its control protein (Con-CHIP) were conducted using a Ni^2+^-nitrilotriacetic acid Sepharose column (Qiagen, Inc., Valencia, CA) and PD-10 desalting column chromatography (GE Healthcare, Piscataway, NJ), respectively, after cell harvest and lysis. The concentration of the end product (Tat-CHIP and Con-CHIP) was measured by the Bradford assay, and the expression of proteins was confirmed by western blotting for His-tag, as described previously^12,30^. The protein bands were visualized using Coomassie blue and chemiluminescent reagents according to the manufacturer’s recommendations (GE Healthcare).

### Toxicity assessment of Tat-CHIP and Con-CHIP in HT22 cells and gerbils

To ensure the safety of Tat-CHIP and Con-CHIP in HT22 cells and gerbils, cell viability was observed using an MTT assay in HT22 cells. In addition, the body temperature of the gerbils was monitored 3 h after Tat-CHIP and Con-CHIP treatment using an infrared thermometer (BiosebLab, Vitrolles, France) to assess the noxious effects.

### Delivery and degradation of Tat-CHIP and Con-CHIP into HT22 cells

Intracellular delivery of Tat-CHIP and Con-CHIP was assessed in HT22 hippocampal cells (ATCC, Manassas, VA). HT22 cells were grown in a dish plate (60 mm diameter) in Dulbecco’s modified Eagle’s medium, as described previously^12,13^. Cells were exposed to various concentrations (0.5–5.0 μM) of Tat-CHIP and Con-CHIP for 1 h or to 5.0 μM of proteins for various times (15–60 min). To detect the degradation of transduced Tat-CHIP, 5.0 μM of Tat-CHIP was added for various times (1–60 h). Thereafter, cells were harvested, and western blot analysis was performed using His-Tag antibody (1:1,000, Santa Cruz Biotechnology, Santa Cruz, CA) and peroxidase-conjugated anti-mouse IgG (1:1,000, Vector, Burlingame, CA), as described previously^13,30^.

### Confirmation of transduced proteins in HT22 cells

Intracellularly delivered proteins were visualized by immunocytochemical staining for His-Tag, as described previously^12,13^. Briefly, cells were grown on coverslips and incubated with 5.0 μM Tat-CHIP and Con-CHIP for 1 h. Thereafter, cells were fixed with 4% paraformaldehyde for 5 min at 25 °C, and immunocytochemical staining was conducted using mouse anti-His-Tag antibody (1:200, Santa Cruz Biotechnology) and Alexa Fluor 488-conjugated anti-mouse IgG (1:1,000, Invitrogen, Carlsbad, CA). Cells were counterstained with 1 μg/mL 4,6-diamidino-2-phenylindole (Roche Applied Science, Mannheim, Germany).

### In vitro effect of Tat-CHIP on hydrogen peroxide-induced oxidative damage in HT22 cells

The optimal concentrations of Tat-CHIP and Con-CHIP proteins to show neuroprotective effects against oxidative stress were determined using the water-soluble tetrazolium salt-1 assay, as described previously^13,30^. Briefly, cells were exposed to 200 μM H_2_O_2_ for 1 h to induce oxidative stress, and various concentrations of Tat-CHIP and Con-CHIP were added to cells with H_2_O_2_ simultaneously. Thereafter, cells were harvested, and cell viability was determined by measuring the optical density of formazan at 450 nm using an enzyme-linked immunosorbent assay (ELISA) microplate reader (Labsystems Multiskan MCC/340, Helsinki, Finland).

The surviving cells, fragmented DNA, and ROS formation were detected by 5-carboxyfluorescein diacetate acetoxymethyl ester (5-CFDA AM, Invitrogen), terminal deoxynucleotidyl transferase dUTP nick end labeling (TUNEL) staining, and 2,7-dichlorofluorescein diacetate (DCF-DA) staining in HT22 cells, respectively, using commercially available kits, as described previously^13,30^. Briefly, cells were exposed to 200 μM H_2_O_2_ for 1 h to induce oxidative stress, and various concentrations of Tat-CHIP and Con-CHIP were added to cells with H_2_O_2_ simultaneously. Thereafter, cells were incubated with 1 μM 5-CFDA AM for 20 min at 37 °C and with 10 μM DCF-DA for 20 min at 37 °C to detect the surviving cells and ROS formation, respectively. TUNEL staining was performed using a cell death detection kit (Roche Applied Science, Basel, Switzerland) according to the manufacturer’s guidelines. 5-CFDA AM-, TUNEL-, and DCF-stained structures were taken using a fluorescence microscope (Nikon Eclipse 80i, Tokyo, Japan), and fluorescence intensity was measured using a Fluoroskan ELISA plate reader (Labsystems Oy, Helsinki, Finland).

### Induction of transient forebrain ischemia in gerbils

Male Mongolian gerbils (6 weeks old) were purchased from Japan SLC Inc. (Shizuoka, Japan), and four animals were housed in type II polycarbonate cages in individually ventilated caging (IVC) systems (Tecniplast®). The handling, use of animals, and experimental protocols were approved by the Institutional Animal Care and Use Committee of Seoul National University (SNU-200313–2-2). All experimental procedures were conducted according to ARRIVE 2.0 guidelines^31^. All methods were performed in accordance with the guidelines from American Veterinary Medical Association and our institution. Transient forebrain ischemia was induced by occlusion of both common carotid arteries for 5 min, as described previously^12,13^. Briefly, animals were anesthetized with 3.0% isoflurane (Baxter, Deerfield, IL) mixed with nitrous oxide and oxygen. Both common carotid arteries were exposed and occluded using aneurysm clips for 5 min, and the interruption of blood flow was confirmed by observing the retinal artery using an ophthalmoscope (HEINE K180®, Heine Optotechnik, Herrsching, Germany). Body temperature was tightly regulated using a rectal probe-regulated thermostat blanket and an infrared radiation lamp. Immediately after reperfusion, Tat-CHIP (0.1, 0.3, or 0.9 mg/kg), Con-CHIP (0.9 mg/kg), or Tat peptide (0.9 mg/kg) was intraperitoneally administered to gerbils. Tat-CHIP, Con-CHIP, and Tat peptide were dissolved in 10% glycerol and normal gerbils were considered as a control group.

### In vivo effect of Tat-CHIP on transient forebrain ischemia in gerbils

One day after ischemia induction, locomotor activity was recorded in the open field cages using a digital camera (Basler, Ahrensburg, Germany) for 60 min. The activity was re-analyzed using XT14 software (EthoVision, Wageningen, The Netherlands) based on the total distance traveled.

Four days after ischemia induction, the animals were re-anesthetized with isoflurane and perfused transcardially with physiological saline and 4% paraformaldehyde. The brain was post-fixed in 4% paraformaldehyde for 24 h at 4 °C and stored in a 30% sucrose solution. Thereafter, the brain was trimmed, and the hippocampal Sects. (30 μm thickness) between 1.4 and 2.0 mm caudal to the bregma^32^ were obtained using a sliding microtome (HM430, Thermo Scientific, Waltham, MA). Four sections, with 120-μm interval from each other, were used for immunohistochemical staining of neuronal nuclei (NeuN), glial fibrillary acidic protein (GFAP), and ionized calcium-binding adapter molecule 1 (Iba-1) to detect the surviving neurons, astrocytes, and microglia in the hippocampus, respectively. Briefly, the sections were sequentially incubated with mouse anti-NeuN antibody (1:1000; EMD Millipore, Temecula, CA), rabbit anti-GFAP antibody (1:1000; EMD Millipore), rabbit anti-Iba-1 antibody (1:500; Wako, Osaka, Japan), biotinylated goat anti-mouse IgG, biotinylated goat anti-rabbit IgG, and streptavidin–peroxidase complex (1:200; Vector). Finally, immunoreactions were developed using 3,3-diaminobenzidine tetrachloride (Sigma, St. Louis, MO).

### In vivo* mechanisms of Tat-CHIP on neuroprotection against ischemia in gerbils*

Three and six hours after ischemia induction, the animals were sacrificed, and both hippocampi were obtained to measure the antioxidant and anti-inflammatory effects of Tat-CHIP against ischemia, respectively. In previous studies, we observed the increases of malondialdehyde (MDA) and pro-inflammatory cytokines in the hippocampus 3 and 6 h after ischemia in gerbils, respectively^33^. MDA, IL-1β, IL-6, and TNF-α levels were measured in the hippocampus using their respective enzyme immunoassay kits (Abcam [Cambridge, UK] for MDA levels and BioSource International Inc. [Camarillo, CA] for IL-1β, IL-6, and TNF-α). In addition, hydroperoxide levels were assessed using the ferrous oxidation-xylenol orange method, as described previously^34^. Levels of glutathione (GSH) and activities of GSH-dependent enzymes including glutathione peroxidase (GPx) and glutathione reductase (GR) were determined in the hippocampus 2 days after ischemia using their ELISA kits (Abcam), as described previously^35^, because depletion of GSH and GSH-dependent enzymes are noted 2 days after ischemia^34,35^.

### Quantification of immunohistochemical data

Four sections, with 120-μm intervals from each other, were used to quantify the data using previously described methods^12,13^. NeuN-immunoreactive neurons were selected at the midpoint of the hippocampal CA1 region, and the number was counted using ImageJ software version 1.80 (National Institutes of Health, Bethesda, MD). In addition, GFAP- and Iba-1-immunoreactive structures were selected, and the background was subtracted. Immunoreactivity was quantified by the summation of each grayscale and pixel number. Data are presented as percentage values compared to the control group values (set at 100%).

### Statistical analyses

All data are shown as the mean ± standard deviation. The differences in means were compared and statistically analyzed using Student’s *t*-test or one-way analysis of variance, followed by Bonferroni’s post-hoc test using GraphPad Prism 5.01 software (GraphPad Software, Inc., La Jolla, CA). Significance was set at *p* < 0.05.

## Results

### Tat-CHIP and Con-CHIP were successfully synthesized

**Figure 1:**
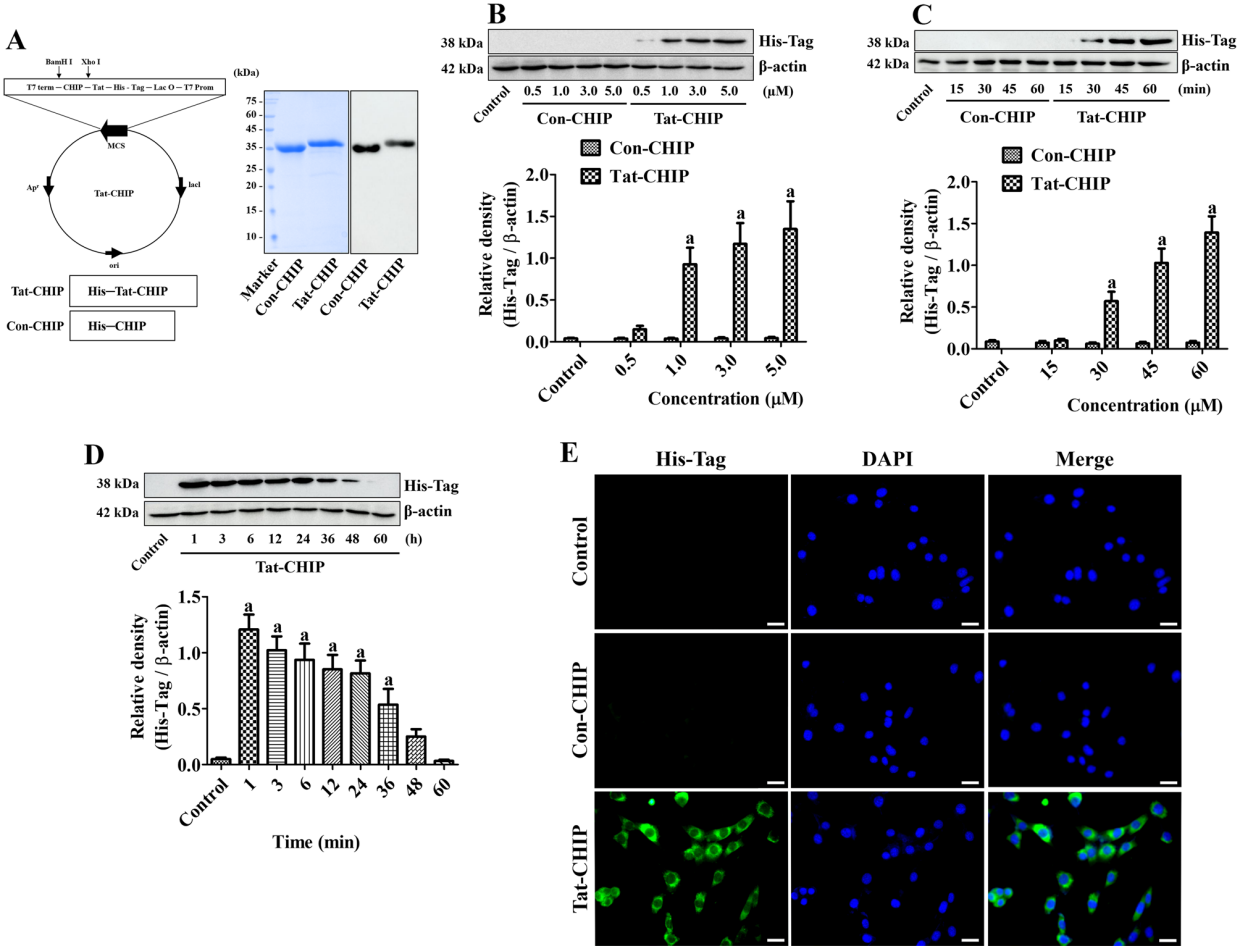
Synthesis of transactivator of transcription-carboxyl-terminus of Hsc70-interacting protein (Tat-CHIP) and its control group (Con-CHIP) and confirmation of their delivery into HT22 cells. **(A)** Tat-CHIP and Con-CHIP plasmids were constructed and cloned in the *Escherichia coli* strain BL21 (DE3) cells. After overexpression, purification, and desalting, protein expression was confirmed by western blotting for His-Tag. **(B** and **C)** Intracellular delivery was confirmed in HT22 cells with various concentrations of Tat-CHIP and Con-CHIP for 1 h and treatment with 5 μM protein for various incubation times, respectively. **(D)** Degradation was observed chronologically after 5 μM Tat-CHIP treatment. **(B–D)** Optical densities of protein bands are expressed as a value of polyhistidine/β-actin. Data were analyzed by Student t-test (^a^*p* < 0.05, significantly different from the control group). All assays were triplicated and the bar graph represents the mean ± standard deviation. **(E)** Intracellular localization of delivered proteins was visualized in HT22 cells by immunocytochemical staining for His-Tag. Scale bar = 20 μm.

Tat-CHIP and Con-CHIP plasmids were cloned in the *E. coli* strain BL21 (DE3) cells, and Tat-CHIP and Con-CHIP proteins were overexpressed, purified, and desalted. The expression of proteins was confirmed by western blotting using a His-tag antibody, and clear single bands were detected at approximately 35 kDa and 38 kDa, respectively (Fig. 1A).

### Confirmation of Tat-CHIP and Con-CHIP toxicity in HT22 cells and gerbils

In HT22 cells, incubation with Tat-CHIP and Con-CHIP did not cause any significant changes in cell viability based on an MTT assay. In addition, treatment with Tat-CHIP and Con-CHIP did not show any significant changes in body temperature in the gerbils 3 h after treatment (Supplementary Fig 1).

### Tat-CHIP, not Con-CHIP, was delivered into HT22 cells

The delivery of Tat-CHIP and Con-CHIP proteins was validated under various concentrations and incubation times in HT22 cells using western blotting for His-Tag. Treatment with Con-CHIP did not show any significant increases in His-Tag protein band at any concentration (0.5–5.0 μM) for 1-h incubation and at 5.0 μM treatment for any incubation time (15–60 min). In contrast, incubation with Tat-CHIP at 1.0 μM or higher concentration for 1 h significantly increased His-Tag levels in HT22 cells. In addition, 5.0 μM of Tat-CHIP for 30 min or longer (by 60 min) significantly increased His-Tag levels in HT22 cells (Fig. 1B and 1C).

Chronological degradation of the delivered Tat-CHIP was confirmed by western blot analysis of His-Tag. Incubation with 5.0 μM of Tat-CHIP for 1 h significantly increased His-Tag levels; thereafter, His-Tag levels decreased in a time-dependent manner. In particular, His-Tag levels remained significantly higher 36 h after treatment (Fig. 1D).

Delivery of Con-CHIP and Tat-CHIP was confirmed morphologically by immunocytochemical staining for His-Tag at 1 h after 5.0 μM protein. In the control and Con-CHIP-treated groups, no His-Tag immunoreactivity was found in the HT22 cells, whereas in the Tat-CHIP group, His-Tag immunoreactivity was confined to the cytoplasm of HT22 cells (Fig. 1E).

### Tat-CHIP ameliorated H_2_O_2_-induced oxidative damage in HT22 cells

**Figure 2:**
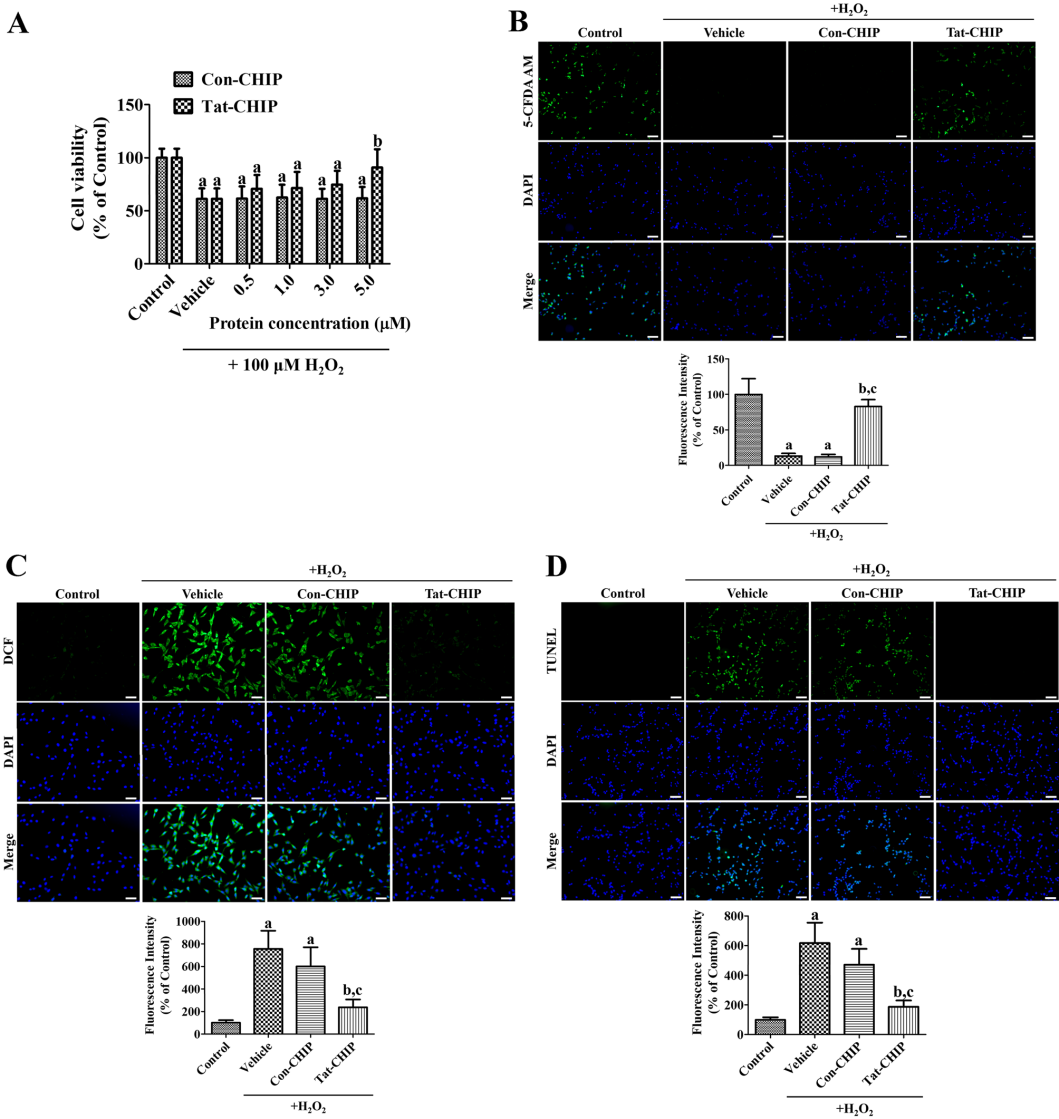
In vitro neuroprotective effects of transactivator of transcription-carboxyl-terminus of Hsc70-interacting protein (Tat-CHIP) and Con-CHIP against oxidative stress induced by 200 μM hydrogen peroxide treatment. **(A)** The cell viability of Tat-CHIP and Con-CHIP was validated after treatment with various concentrations of proteins for 1 h using water-soluble tetrazolium salt-1 assay. **(B–D)** Surviving neurons, reactive oxygen species formation, and fragmented DNA were observed using 5-carboxyfluorescein diacetate acetoxymethyl ester (5-CFDA AM), 2,7-dichlorofluorescein diacetate (DCF), and terminal deoxynucleotidyl transferase dUTP nick end labeling (TUNEL) staining, respectively. Scale bar = 50 μm. The fluorescence intensities of 5-CFDA AM-, DCF-, and TUNEL-stained structures were determined spectrophotometrically. **(A–D)** Data were analyzed by one-way analysis of variance, followed by a Bonferroni’s post-hoc test (^a^*p* < 0.05, significantly different from the control group; ^b^*p* < 0.05, significantly different from the vehicle-treated group; ^c^*p* < 0.05, significantly different from the Con-CHIP-treated group). All assays were triplicated and the bar graph represents the mean ± standard deviation.

Oxidative stress induced by 200 μM H_2_O_2_ significantly decreased the viability of HT22 cells to 61.3% of the control group. Treatment with Con-CHIP did not show any significant improvements in cell viability in HT22 cells with 5.0 μM protein, whereas incubation with Tat-CHIP significantly mitigated the reduction of cell viability at 5.0 μM of protein treatment to 90.8% of the control group (Fig. 2A).

The surviving cells were visualized by 5-CFDA AM staining in HT22 cells. In the control group, 5-CDFA AM-stained cells were abundant in the HT22 cells, whereas in the vehicle- and Con-CHIP-treated groups, 5-CDFA AM-stained cells nearly disappeared, and the fluorescence intensities were 13.2% and 12.1% of the control group, respectively. In the Tat-CHIP-treated group, many 5-CDFA AM-stained cells were found in HT22 cells, and the fluorescence intensity was significantly higher (82.9% of the control group) than in the vehicle- and Con-CHIP-treated groups (Fig. 2B).

ROS induced by 200 μM H_2_O_2_ was visualized using DCF staining in HT22 cells. In the control group, DCF staining was significantly faintly detected in a few HT22 cells. In the vehicle-treated group, DCF-stained cells were abundantly observed, and fluorescence intensity was significantly increased to 756.0% of the control group. In the Con-CHIP-treated group, DCF fluorescence intensity was slightly lower than that in the vehicle-treated group. In the Tat-CHIP-treated group, significantly lower DCF fluorescence intensity (238.3% of control) was observed compared to the vehicle- and Con-CHIP-treated groups (Fig. 2C).

DNA fragmentation induced by 200 μM H_2_O_2_ was visualized by TUNEL staining. In the control group, few TUNEL-positive cells were found in HT22 cells, whereas in the vehicle- and Con-CHIP-treated groups, TUNEL-positive cells were abundant, and their fluorescence intensities were significantly increased to 617.1% and 471.3% of the control group, respectively. In the Tat-CHIP-treated group, few TUNEL-positive cells were found, and the fluorescence intensity was significantly lower (186.7% of control) compared to those of the vehicle- and Con-CHIP-treated groups (Fig. 2D).

### Tat-CHIP alleviated ischemia-induced hyperlocomotion and neuronal death in the hippocampus

**Figure 3:**
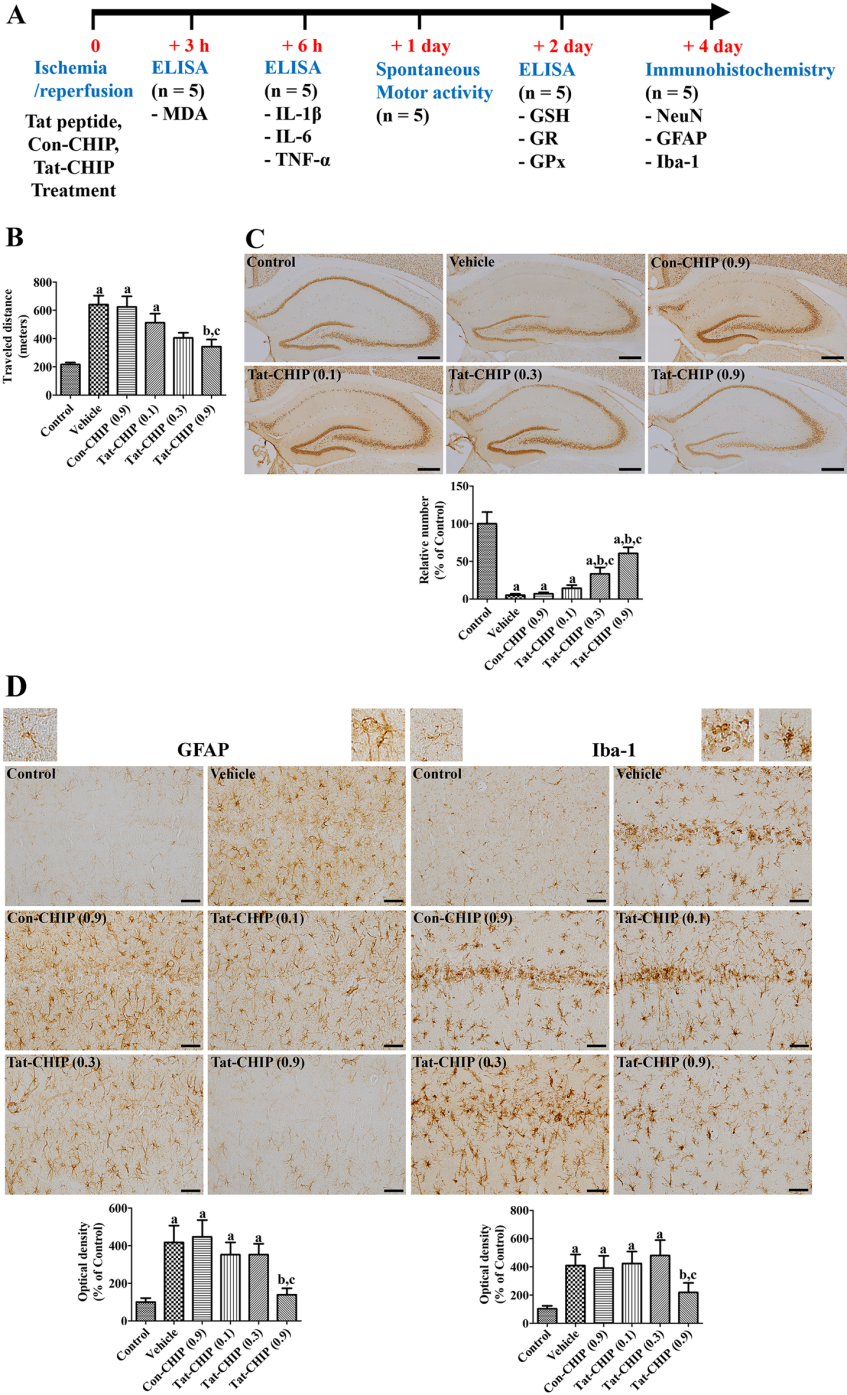
In vivo neuroprotective effects of transactivator of transcription-carboxyl-terminus of Hsc70-interacting protein (Tat-CHIP) and Con-CHIP against ischemic damage induced by 5 min of transient forebrain ischemia in gerbils. **(A)** Experimental design of animal study is indicated. **(B)** Locomotion was recorded, and the distance traveled was analyzed 1 day after ischemia induction. **(C)** Mature neurons in the hippocampus were detected by immunohistochemical staining for neuronal nuclei (NeuN) 4 days after ischemia induction. The number of NeuN immunoreactive neurons in CA1 region was counted and is represented as a percentile value versus control group. Scale bar = 400 μm. **(D)** Astrocytes and microglia were observed by immunohistochemical staining for glial fibrillary acidic protein (GFAP) and ionized calcium-binding adapter molecule 1 (Iba-1) in the CA1 region 4 days after ischemia induction. Highly magnified inset images were also demonstrated in control and vehicle-treated groups. Scale bar = 50 μm. Optical densities were measured and are represented as a relative percentile value of GFAP and Iba-1 immunoreactivity versus control group per section, respectively. **(B–D)** Data were analyzed by one-way analysis of variance, followed by a Bonferroni’s post-hoc test (n = 5 per group; ^a^*p* < 0.05, significantly different from the control group; ^b^*p* < 0.05, significantly different from the vehicle-treated group; ^c^*p* < 0.05, significantly different from the Con-CHIP-treated group). The bar graph represents the mean ± standard deviation.

The neuroprotective effects of Tat-CHIP were evaluated by measuring locomotion 1 day after ischemia induction (Fig. 3A), because hyperlocomotion can be used as a predictor of CA1 damage ^36,37^. In the vehicle- and Con-CHIP-treated groups, the traveled distance was significantly increased to 295.3% and 287.6% of the control group, respectively. In the Tat-CHIP-treated group, the traveled distance decreased in a concentration-dependent manner, and the traveled distance was significantly shorter in 0.9 mg/kg Tat-CHIP-treated group (157.9% of control) than in the vehicle- or Con-CHIP-treated groups (Fig. 3B).

The neuroprotective effects of Tat-CHIP were confirmed by visualization of surviving cells 4 days after ischemia induction. In the control group, NeuN-immunoreactive neurons were observed in the hippocampus. In the vehicle-treated group, NeuN-immunoreactive neurons were few in the CA1 region, whereas other regions showed similar morphology to the control group. In this group, the number of NeuN-immunoreactive neurons was 5.1% of the control group. In the Con-CHIP-treated group, the distribution pattern of NeuN-immunoreactive neurons was similar to that of the vehicle-treated group, and the number was 6.8% of the control group. In the Tat-CHIP-treated groups, NeuN-immunoreactive neurons were increased in the CA1 region in a dose-dependent manner. In the 0.9 mg/kg Tat-CHIP-treated group, 60.6% of the control group had NeuN-immunoreactive neurons (Fig. 3C).

### Tat-CHIP inhibited the activation of astrocytes and microglia in the hippocampus after ischemia induction

The activation of astrocytes and microglia induced by ischemia was visualized by immunohistochemical staining for GFAP and Iba-1, respectively, 4 days after ischemia induction. GFAP-immunoreactive astrocytes showed a similar distribution pattern in all groups, whereas Iba-1 immunoreactive microglia were aggregated in the stratum pyramidale due to neuronal death. In the control group, GFAP-immunoreactive astrocytes and Iba-1 immunoreactive microglia were detected in the stratum radiatum and stratum oriens of the CA1 region. Their cytoplasm was small, and they had long and thin processes (resting form) as shown in inset images in Fig. 3D. In the vehicle-treated group, GFAP-immunoreactive astrocytes and Iba-1 immunoreactive microglia located in the stratum oriens and radiatum had hypertrophied cytoplasm with thickened processes (activated form) as shown in inset images in Fig. 3D. In addition, Iba-1 immunoreactive microglia in the stratum pyramidale had round or ameboid cytoplasm without processes (phagocytic form) as shown in an inset image in Fig. 3D. In this group, GFAP and Iba-1 immunoreactivities were significantly increased to 417.3% and 409.3% of the control group, respectively. In the Con-CHIP-treated group, the distribution pattern and immunoreactivity of GFAP and Iba-1 were similar to those in the vehicle-treated group. In the Tat-CHIP-treated groups, GFAP-immunoreactive astrocytes were mixed with resting and activated forms, whereas the phagocytic form of Iba-1 immunoreactive microglia in the stratum pyramidale decreased in a dose-dependent manner. In particular, GFAP and Iba-1 immunoreactivities in the 0.9 mg/kg Tat-CHIP-treated group showed significantly lower levels (138.7% and 219.3%, respectively) than those in the vehicle- and Con-CHIP-treated groups (Fig. 3D).

### Tat-CHIP mitigated ischemia-induced oxidative stress in gerbils

**Figure 4:**
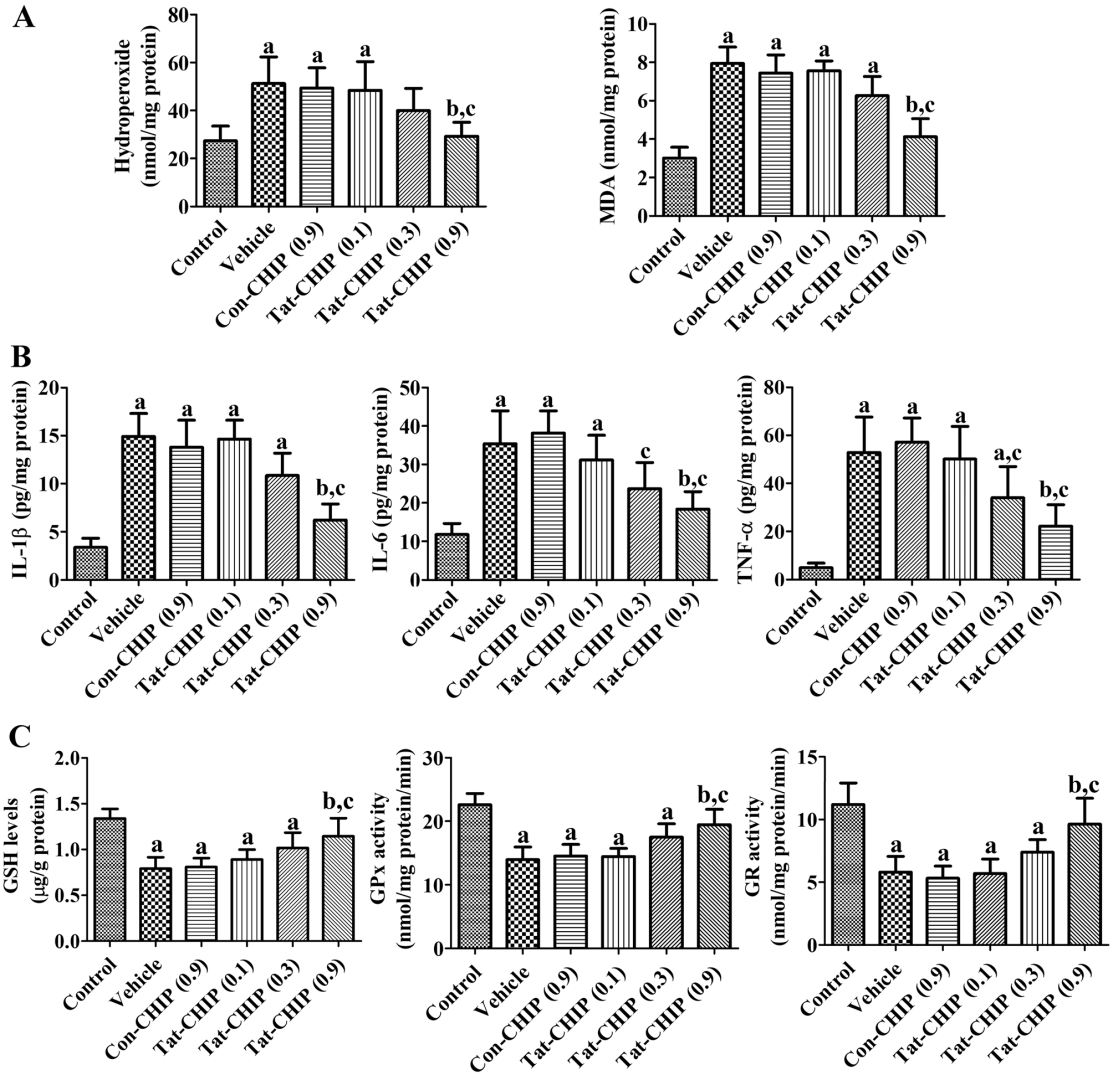
In vivo neuroprotective mechanisms of transactivator of transcription-carboxyl-terminus of Hsc70-interacting protein (Tat-CHIP) against ischemic damage in gerbils. **(A)** Markers for oxidative stress, such as hydroperoxides and malondialdehyde (MDA) levels, were measured in the gerbil hippocampus 3 h after ischemia induction. **(B)** Pro-inflammatory cytokines, such as interleukin (IL)-1β, IL-6, and tumor necrosis factor-α (TNF-α) were assessed in the gerbil hippocampus 6 h after ischemia induction. **(C)** Glutathione (GSH) and its redox enzymes such as glutathione peroxidase (GPx) and glutathione reductase were determined in the gerbil hippocampus 2 days after ischemia induction. **(A–C)** Data were analyzed by one-way analysis of variance, followed by a Bonferroni’s post-hoc test (n = 5 per group; ^a^*p* < 0.05, significantly different from the control group; ^b^*p* < 0.05, significantly different from the vehicle-treated group; ^c^*p* < 0.05, significantly different from the Con-CHIP-treated group). The bar graph represents the mean ± standard deviation.

The antioxidant effects of Tat-CHIP were evaluated by measuring the hydroperoxide and MDA levels in the hippocampus 3 h after ischemia induction. In the vehicle-treated group, hydroperoxide and MDA levels were significantly increased to 187.4% and 263.8% of the control group, respectively. In the Con-CHIP-treated group, hydroperoxide and MDA levels were similar to those in the vehicle-treated group. In the Tat-CHIP-treated group, hydroperoxide and MDA levels decreased in a dose-dependent manner. Specifically, hydroperoxide and MDA levels were significantly lower (107.2% and 137.0% of the control group, respectively) in the 0.9 mg/kg Tat-CHIP-treated group compared to the vehicle- and Con-CHIP-treated groups (Fig. 4A).

### Tat-CHIP ameliorated the increase in pro-inflammatory cytokines in gerbils after ischemia induction

The anti-inflammatory effects of Tat-CHIP were assessed in the hippocampus 6 h after ischemia induction by measuring pro-inflammatory cytokine levels, such as IL-1β, IL-6, and TNF-α. In the vehicle-treated group, IL-1β, IL-6, and TNF-α levels were significantly increased to 442.3%, 301.3%, and 1061.6% of the control group, respectively. In the Con-CHIP-treated group, similar levels of IL-1β, IL-6, and TNF-α were observed in the hippocampus. In the Tat-CHIP-treated group, IL-1β, IL-6, and TNF-α levels were decreased in a dose-dependent manner and were significantly lower in the 0.9 mg/kg Tat-CHIP-treated group (184.5%, 156.4%, and 446.8% of the control group, respectively) than those in the vehicle- and Con-CHIP-treated groups (Fig. 4B).

### Tat-CHIP alleviates the reduction of GSH level, GPx, and GR activities in gerbils after ischemia induction

The effects of Tat-CHIP on GSH and its redox enzymes were assessed in the hippocampus 2 days after ischemia by determination of GSH level, GPx, and GR activities. In the vehicle-treated group, GSH level, GPx, and GR activities were significantly decreased to 59.0%, 61.7%, and 51.8% of control group, respectively. In the Con-CHIP-treated group, GSH and its redox enzymes showed similar levels compared to that in its respectively vehicle-treated group. In the Tat-CHIP-treated group, GSH level, GPx, and GR activities were concentration-dependently increased and their level or activities showed significant increases in 0.9 mg/kg Tat-CHIP-treated group compared to those in vehicle-treated group, which were 85.5%, 86.1%, and 86.0% of control group (Fig. 4C).

## Discussion

CHIP is believed to be a key player in neurological disorders because it has E3 ubiquitin ligase activity to remove damaged or toxic proteins, and is highly expressed in the brain tissue^14,38^. CHIP levels are increased in mouse peri-infarct tissues after middle cerebral artery occlusion^39^ and in postmortem samples from ischemic patients^21^. In the present study, we synthesized a Tat-CHIP fusion protein to facilitate the crossing of the blood–brain barrier and to deliver the proteins into neurons because gene delivery is difficult in clinical practice. Tat-CHIP can be delivered in a concentration- and time-dependent manner, and we confirmed the cytosolic expression of the delivered Tat-CHIP protein by visualization of the His-Tag, which was inserted into the plasmid. Tat fusion protein can cross the plasma membrane without a delivery vehicle, although its entry mechanism is not fully understood^40^. The Tat peptide is most widely used as a protein transduction domain and is applicable to the transduction of double-stranded RNA binding protein and short interfering RNA^41,42^.

In this study, we observed the effects of CHIP on oxidative damage induced by H_2_O_2_ treatment in HT22 cells using Tat-CHIP. We observed cell viability to determine the optimal concentration of Tat-CHIP against oxidative damage, and 5.0 μM Tat-CHIP significantly improved cell viability in HT22 cells after H_2_O_2_ treatment. This result was consistent with that of a previous study showing that CHIP overexpression protected N2a cells from oxygen-glucose deprivation^24^, whereas silencing of CHIP aggravated the cell death induced by oxygen-glucose deprivation^22,25^. In organotypic slice culture, overexpression of CHIP significantly ameliorated endoplasmic reticulum stress-induced DNA fragmentation and apoptotic cell death in the hippocampus^43^. In addition, overexpression and depletion of CHIP promote and suppress cell proliferation in brain microvascular endothelial cells, respectively, after stretch-induced injury^44^. However, conflicting evidence has also demonstrated that CHIP overexpression in HT22 cells worsens cell damage after glutamate treatment, whereas downregulation of CHIP promotes cell survival of cells after oxygen–glucose deprivation in primary neuronal culture^21^.

To elucidate the roles of Tat-CHIP against oxidative or ischemic damage, we investigated the effects of Tat-CHIP against ischemic damage in gerbils, which is a convenient ischemic model only by occlusion of both common carotid arteries due to the absence of the posterior communicating arteries^45^. We measured the distance traveled 1 day after ischemia induction because functional impairments of the CA1 region, not other susceptible regions such as the striatum and cortex, cause hyperlocomotion at this time^36,37^. Administration of Tat-CHIP dose-dependently ameliorated ischemia-induced hyperlocomotion 1 day after ischemia induction and improved neuronal survival in the CA1 region 4 days after ischemia induction based on immunohistochemical staining for NeuN. Several studies have demonstrated that overexpression of CHIP protects neurons against ischemic damage induced by middle cerebral artery occlusion^23,39^. CHIP overexpression attenuates cellular damage in the heart after cardiac ischemia^46^. In addition, CHIP overexpression mitigates neuronal damage against traumatic brain injury^44^ and Alzheimer’s disease^47,48^. All the studies used genetic overexpression of CHIP, which is difficult to apply to humans owing to safety concerns, and in the present study, we synthesized Tat-CHIP to facilitated clinical application.

Transient forebrain ischemia causes neuronal damage through various mechanisms, and the most reliable cell death mechanisms are oxidative stress and inflammation in the CA1 region. Ischemia activates astrocytes and microglia, which release large amounts of pro-inflammatory cytokines, such as IL-1β, IL-6, and TNF-α^3,5^, and produce ROS by nicotinamide adenine dinucleotide phosphate oxidase^4^. In this study, we visualized astrocytes and microglia in the hippocampal CA1 region 4 days after ischemia induction. Ischemic insults cause morphological changes in astrocytes and microglia and aggregation of microglia in damaged regions^49–51^. Treatment with Tat-CHIP, but not Con-CHIP, decreased the activation of astrocytes and microglia in a concentration-dependent manner. In addition, transient forebrain ischemia significantly increased hydroperoxide and MDA levels after ischemia induction and Tat-CHIP, but not Con-CHIP, mitigating the increases in hydroperoxides and MDA levels in a dose-dependent manner. Previous studies have shown that CHIP insufficiency increased lipid oxidation^22,52^, enhanced sensitivity to membrane damage by digitonin^53^, and decreased GSTA4 levels^25^, which quenches lipid peroxidation after oxidative stress^54^. Pro-inflammatory cytokines, such as IL-1β, IL-6, and TNF-α, were significantly increased in the hippocampus after ischemia induction, and Tat-CHIP attenuated the increase in pro-inflammatory cytokine release, suggesting the anti-inflammatory potential of Tat-CHIP in ischemic damage. This result was consistent with that of a previous study showing that CHIP overexpression reduced IL-1β, IL-6, and TNF-α levels in brain tissues after middle cerebral artery occlusion^39^.

Transient forebrain ischemia depleted the GSH and its redox enzymes in the hippocampus, which is the main causes of neuronal death^55^. In contrast, GSH acts as a major intracellular antioxidant and reduces oxidative neuronal damage against ischemic damage^56^. In the present study, we measured GSH level, GPx, and GR activities in the hippocampus 2 days after ischemia because GSH depletion is most prominent 2 days after ischemia in gerbils^34,57^. Consistently with previous studies, GSH level, GPx, and GR activities were significantly decreased in the hippocampus 2 days after ischemia and administration with 0.9 mg/kg Tat-CHIP, not Con-CHIP, significantly alleviated the reduction of GSH level, GPx, and GR activities. These results suggest that Tat-CHIP treatment showed neuroprotective actions by maintaining GSH and its redox enzymes in the hippocampus.

The limitation of this study is the narrow time window used. We treated Tat-CHIP protein immediately after reperfusion and tried to elucidate the neuroprotective effects of Tat-CHIP against ischemia in delayed treatment of Tat-CHIP 3–12 h after ischemia to alleviate the neuronal damage in the hippocampal CA1 region after ischemia.

In conclusion, Tat-CHIP protects neurons from oxidative stress and ischemic damage by downregulating oxidative damage and neuroinflammation as well as maintaining GSH and its redox enzymes. Protein therapy using Tat-CHIP can be an option to reduce neuronal damage in the early period of ischemia.

## Supplementary Information

Supplementary Information 1.

Supplementary Information 2.

## Data Availability

The datasets used and/or analysed during the current study available from the corresponding author on reasonable request.
