# Supplementary material for: CHIP ameliorates neuronal damage in H2O2-induced oxidative stress in HT22 cells and gerbil ischemia
Source: Sci Rep. 2022 Nov 30;12:20659. doi: 10.1038/s41598-022-22766-0 (PMC9712579; doi:10.1038/s41598-022-22766-0)
Supplement: Supplementary file 1 — Supplementary Information 1. [file 41598_2022_22766_MOESM1_ESM.pdf]

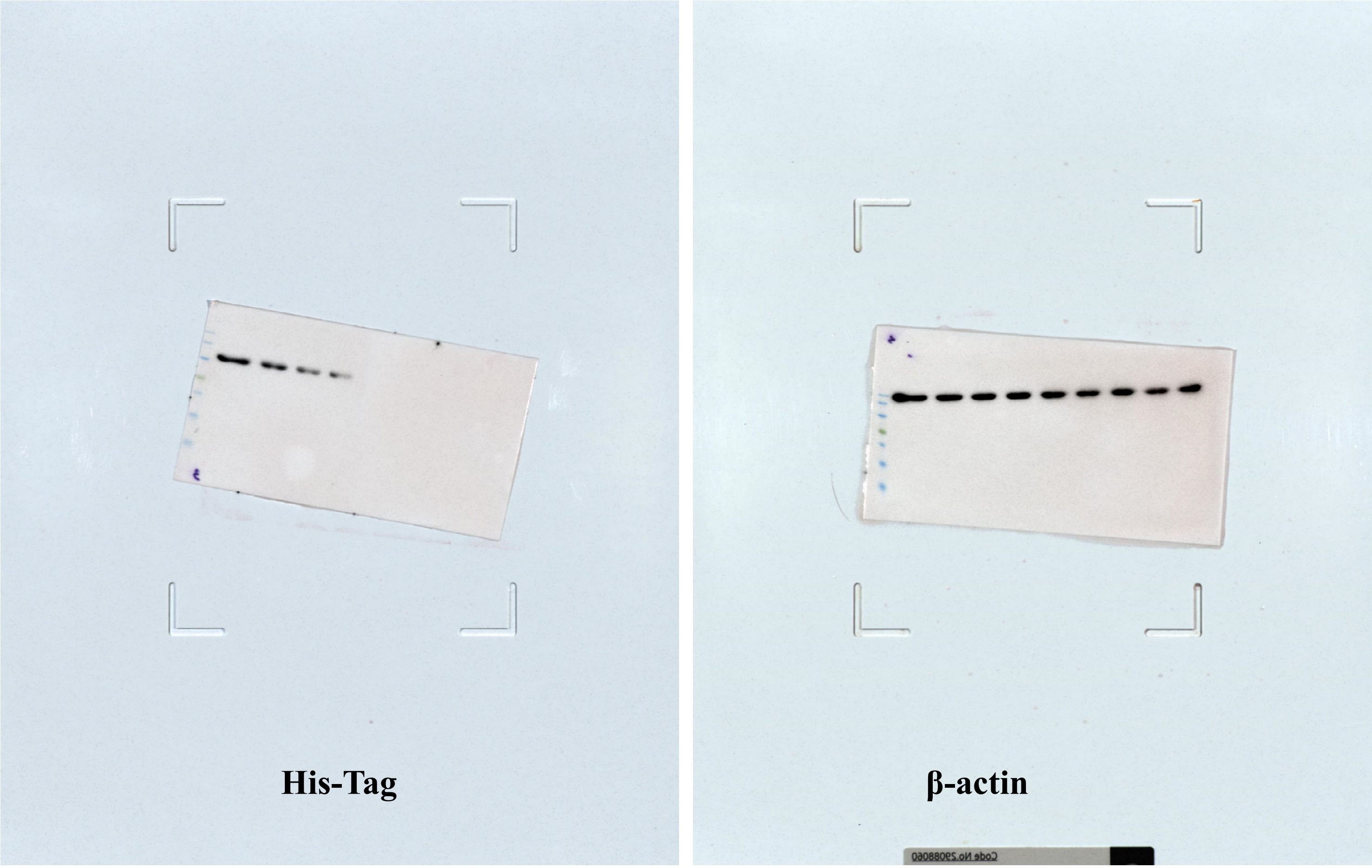

Control 0.5 1.0 3.0 5.0 0.5 1.0 3.0 5.0 (μM)

Con-CHIP Tat-CHIP

Figure 1A

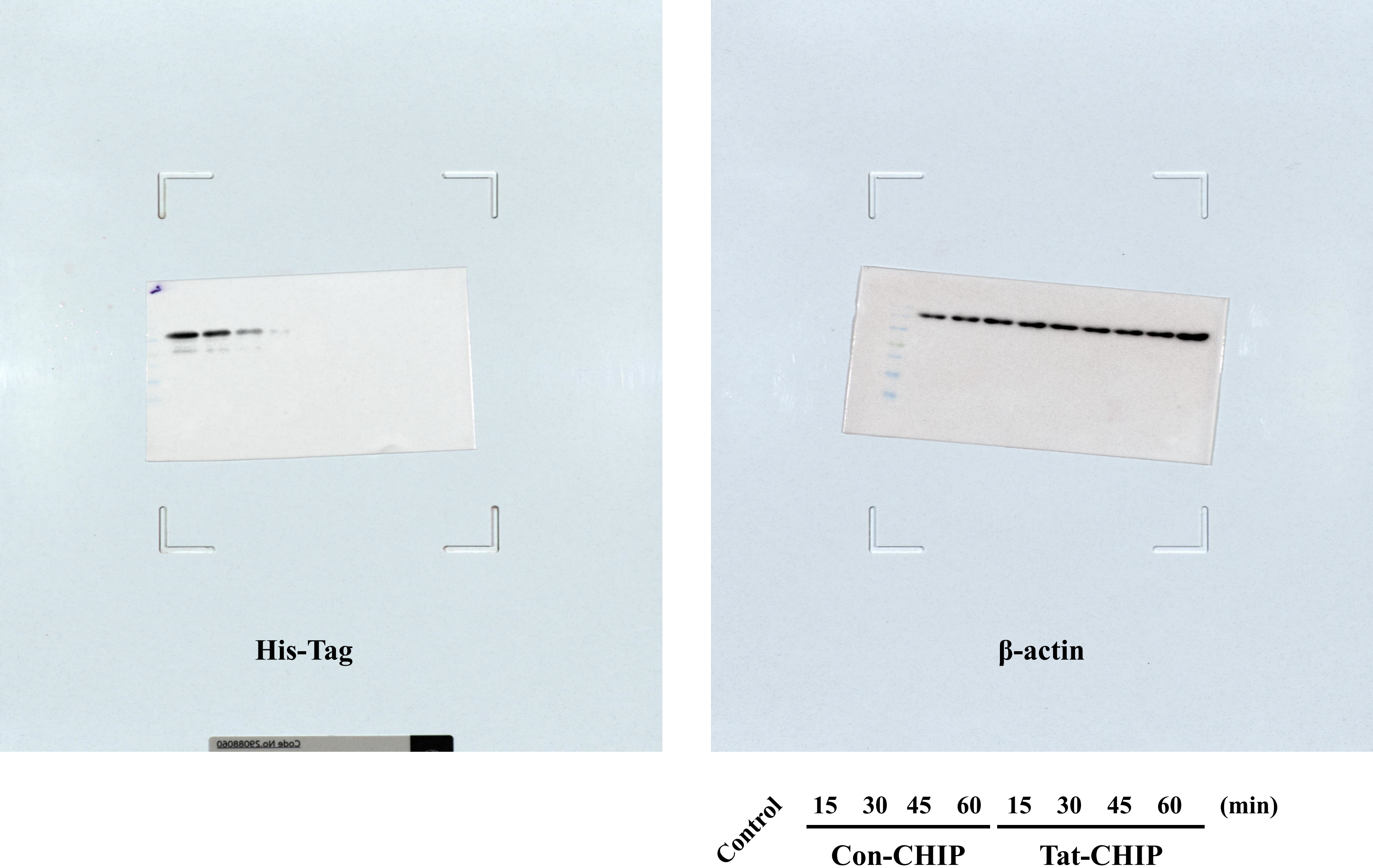
His-Tag
β-actin
Control
15 30 45 60
Con-CHIP
15 30 45 60
Tat-CHIP
(min)

Figure 1B

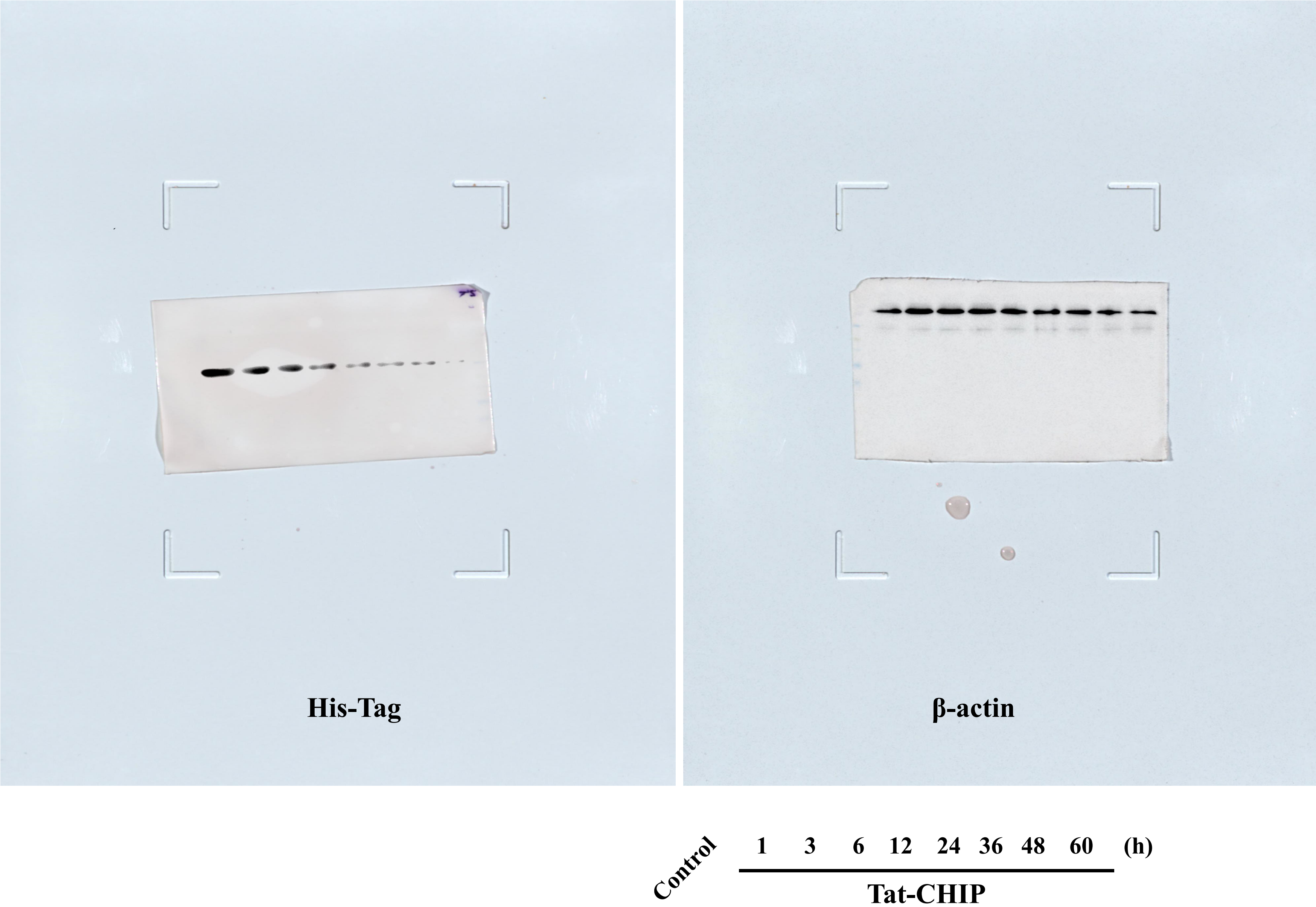

Figure 1C
