# Supplementary material for: CHIP ameliorates neuronal damage in H2O2-induced oxidative stress in HT22 cells and gerbil ischemia
Source: Sci Rep. 2022 Nov 30;12:20659. doi: 10.1038/s41598-022-22766-0 (PMC9712579; doi:10.1038/s41598-022-22766-0)

**Supplementary Figure Legends**

**Supplementary Figure 1.** Toxicity of transactivator of transcription-carboxyl-terminus of Hsc70-interacting protein (Tat-CHIP) and its control group (Con-CHIP) in HT22 cells and gerbils. **(A)** Cellular damage was assessed by MTT assays with different concentrations of Tat-CHIP and Con-CHIP for 1 h. **(B)** Body temperature was monitored after intraperitoneal injection of Tat-CHIP and Con-CHIP by 3 h after treatment. There were no significant differences among groups.


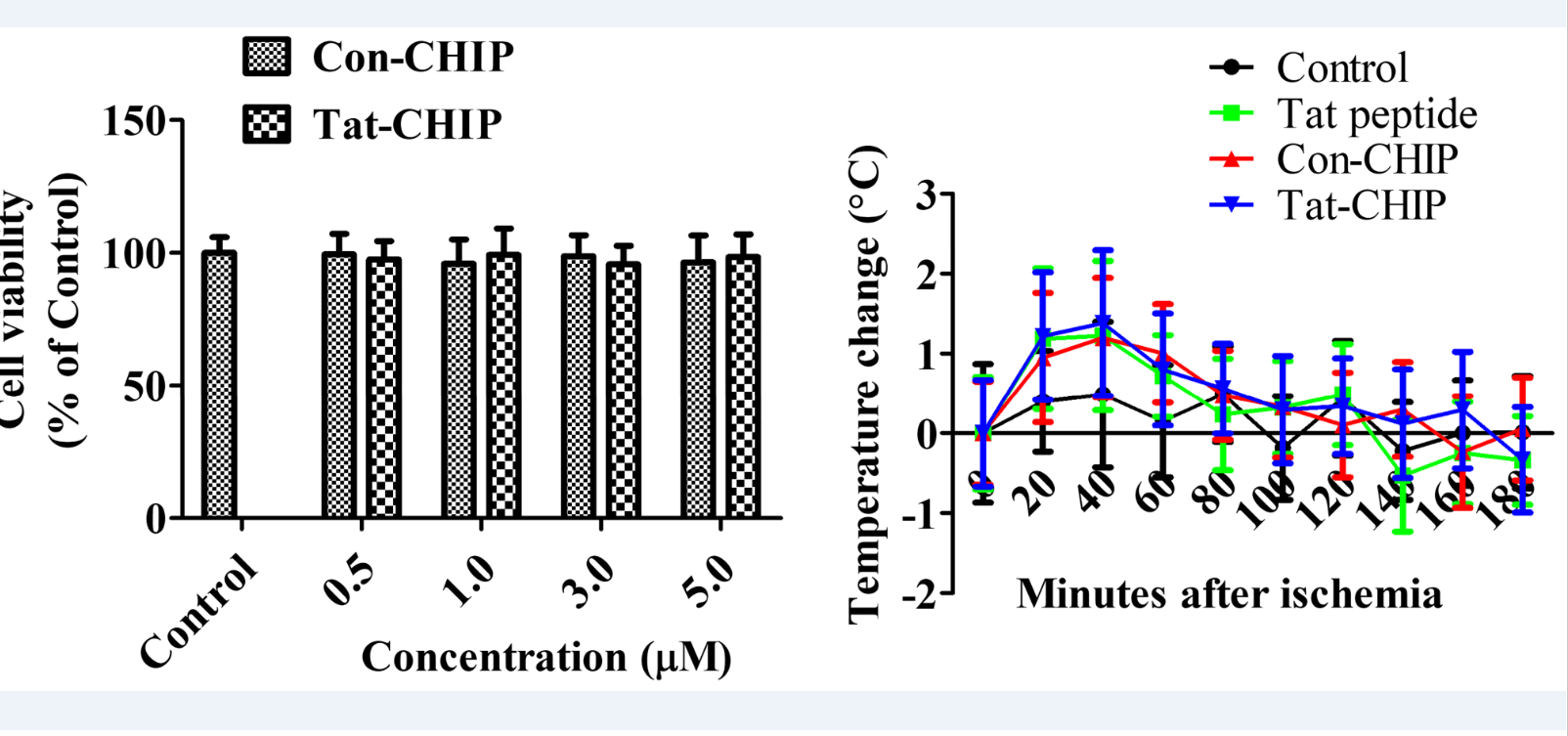

Supplement: Supplementary file 2 — Supplementary Information 2. [file 41598_2022_22766_MOESM2_ESM.docx]
